# Die aktuelle Datenlage zur sexuellen Gesundheit im deutschsprachigen Raum

**DOI:** 10.1007/s00103-021-03442-6

**Published:** 2021-10-26

**Authors:** Ursula von Rüden, Angelika Heßling, Uwe Koch-Gromus

**Affiliations:** 1 Abteilung Q – Forschung, Zielgruppen, Lebenslagen, Referat Q3 – Evaluation, Methoden, Forschungsdaten, Bundeszentrale für gesundheitliche Aufklärung (BZgA), Maarweg 149–161, 50825 Köln, Deutschland; 2 Abteilung S – Sexualaufklärung, Verhütung und Familienplanung, Referat S3 – Aufgabenkoordinierung, Nationale und internationale Zusammenarbeit, Forschung und Fortbildung, Bundeszentrale für gesundheitliche Aufklärung (BZgA), Maarweg 149–161, 50825 Köln, Deutschland; 3 Institut und Poliklinik für Medizinische Psychologie, Universitätsklinikum Hamburg-Eppendorf, Martinistraße 52, 20246 Hamburg, Deutschland

Sexuelle Gesundheit ist eines der 5 Kernthemen der globalen Strategie der Weltgesundheitsorganisation (WHO) zur reproduktiven Gesundheit und wird wie folgt definiert: „Sexuelle Gesundheit ist der Zustand körperlichen, emotionalen, geistigen und sozialen Wohlbefindens bezogen auf die Sexualität und bedeutet nicht nur die Abwesenheit von Krankheit, Funktionsstörungen und Schwäche. Sexuelle Gesundheit erfordert sowohl eine positive, respektvolle Herangehensweise an Sexualität und sexuelle Beziehungen als auch die Möglichkeit für lustvolle und sichere sexuelle Erfahrungen, frei von Unterdrückung, Diskriminierung und Gewalt. Wenn sexuelle Gesundheit erreicht und bewahrt werden soll, müssen die sexuellen Rechte aller Menschen anerkannt, geschützt und eingehalten werden“ [1].

Fragen zur sexuellen Gesundheit sind weitreichend und umfassen sexuelle Orientierung und Geschlechtsidentität, sexuelle Verhaltensweisen, Beziehungen und Vergnügen. Sie umfassen aber auch negative Aspekte oder Infektionen z. B. mit HIV oder andere sexuell übertragbare Infektionen (STI) oder Infektionen des Reproduktionstrakts (RTI) sowie deren Folgen wie etwa Krebs und Unfruchtbarkeit. Themen wie unbeabsichtigte Schwangerschaften, Schwangerschaftsabbrüche, sexuelle Funktionsstörungen und sexualisierte Gewalt gehören ebenfalls zur sexuellen Gesundheit.

Aufgrund gesellschaftlicher Debatten zu den Themen HIV und ungewollte Schwangerschaften, aber auch der Forderung nach evidenzbasierter Prävention und Sexualaufklärung sowie der Diskussion um Diversität und des Themenschwerpunkts Prävention von sexualisierter Gewalt hat die sozialwissenschaftliche Forschung zur sexuellen Gesundheit in den letzten Jahren viele Studien und Ergebnisse für die Bundesrepublik Deutschland hervorgebracht. Um diese neuen Erkenntnisse zeitnah in die Fachöffentlichkeit zu transferieren und weitere Forschungsbemühungen in diesem Bereich anzuregen, wurde das hier vorgelegte Themenheft „Sexuelle Gesundheit“ konzipiert. Es soll den Themenkomplex der sexuellen Gesundheit in seinen vielfältigen Zusammenhängen abbilden.

Im ersten Teil des Themenheftes werden sexualwissenschaftliche Ergebnisse für Erwachsene und Jugendliche/junge Erwachsene in Deutschland dargestellt, für die mit dem GeSiD-Projekt (Gesundheit und Sexualität in Deutschland) und mit der Repräsentativbefragung „Jugendsexualität“ eine aktuelle und bevölkerungsrepräsentative Datenbasis zur Verfügung steht.

Im einleitenden Beitrag geben* P. Briken et al.* einen knappen Überblick über Zielsetzungen, Methodik und Untersuchungsgruppe der GeSiD-Studie, in der im Rahmen eines von der BZgA geförderten Projektes ein breites Spektrum von Fragestellungen der Sexualität im Erwachsenenalter auf der Basis von knapp 5000 interviewten Personen untersucht wird. Diese Studie bildet die Grundlage der drei nachfolgenden Beiträge.

Zunächst analysieren *F. Brunner et al.* Daten zur Lebenszeitprävalenz des Erlebens von sexueller Gewalt und deren Abhängigkeit von ausgewählten soziodemografischen wie gesundheitsbezogenen Faktoren. Die Studie deckt wichtige Zusammenhänge von sexueller Gewalt mit psychischer und somatischer Gesundheit auf.

*S. Matthiesen et al. *gehen danach auf der Basis des GeSiD-Datensatzes der Frage nach, wie sich der Wissensstand in der deutschen Erwachsenenbevölkerung zu sexuell übertragbaren Infektionen (STI) darstellt. Die Autoren/innen sehen hierin eine wichtige Grundlage für eine zielgruppenspezifische Präventionsarbeit bei STI.

Ganz anders nutzen *C. Muschalik et al.* die GeSiD-Daten: Sie diskutieren am Beispiel dieser Erhebung die Notwendigkeit bzw. Möglichkeiten einer Abkehr von dem aus ihrer Sicht veralteten Verständnis von Geschlecht als „binäre“ Variablen (weiblich/männlich). Neben der Einführung einer weiteren Kategorie „divers“ für das Geschlecht sehen sie in der Verwendung weiterer Parameter wie etwa „sexuelle Anziehung“ Möglichkeiten für eine angemessenere Operationalisierung von Geschlecht in (repräsentativen) Befragungen.

Die beiden nachfolgenden Artikel stützen sich auf die jüngste Erhebungswelle der seit knapp 40 Jahren regelmäßig von der BZgA durchgeführten Repräsentativbefragung „Jugendsexualität“. Hier wurden mehr als 6000 Jugendliche und junge Erwachsene in Deutschland befragt. Im ersten der beiden Beiträge beschreiben und analysieren *S. Scharmanski *und* A. Heßling* das Sexual- und Verhütungsverhalten der Zielgruppe. Der Vergleich mit vorangegangenen Erhebungswellen weist auf einige bemerkenswerte Veränderungen im Sexualverhalten Jugendlicher in Deutschland hin.

*C. Erkens et al.* nutzen die aktuellen Daten der Studie „Jugendsexualität“ für eine Analyse der Prävalenzen erlebter körperlicher und nicht körperlicher sexualisierter Gewalt in der Erfahrung junger Menschen. Weitere Inhalte des Beitrags beziehen sich auf die Täterkreise und das Disclosure-Verhalten (Offenbarungsverhalten) Betroffener.

Eine andere Form des Missbrauchs von Kindern und Jugendlichen behandelt der Beitrag von *A. Dekker et al. *Sie untersuchen auf der Grundlage einer Befragung von Schulleitungen die nichtkonsensuelle Weiterleitung persönlicher erotischer Fotos mittels digitaler Medien und deren Folgen in Schleswig-Holstein. Sie sehen in ihren Ergebnissen ein sexualbezogenes Gesundheitsproblem erheblichen Ausmaßes, das die Notwendigkeit spezifischer Präventionsmaßnahmen erfordert.

Die beiden folgenden Beiträge befassen sich mit dem Problem „ungewollt schwanger werden“. Zunächst untersuchen *B. Paetzel et al. *an Schülerinnen und Schülern die Akzeptanz und Wirksamkeit ärztlicher Interventionen auf Wissen und Selbstwirksamkeitserleben bei diesem Thema. Die Ergebnisse zeigen, dass schulische Maßnahmen der Primär- und Sekundärprävention erfolgreich einer möglichen ungeplanten Schwangerschaft vorbeugen können.

Anschließend analysieren *C. Helfferich et al. *auf der Basis von Befragungsdaten zu 17.400 Schwangerschaften aus der BZgA-Studie „frauen leben 3. Familienplanung im Lebenslauf“ die Gründe, warum Frauen, die schwanger werden können und aktuell keinen Kinderwunsch haben, nicht verhüten und damit eine unbeabsichtigte Schwangerschaft riskieren. Die Ergebnisse weisen auf ein multifaktorielles Motivationsgeschehen hin.

Die nachfolgenden Beiträge behandeln unterschiedliche aktuelle sexualwissenschaftliche Themen und basieren auf digitalen Zugängen.

*N. Döring *und* M. Conde *arbeiten mittels eines Scoping-Reviews den internationalen Forschungsstand zu sexuellen Gesundheitsinformationen in sozialen Medien auf. Dies geschieht unter der Perspektive, sexuelle und reproduktive Gesundheitsinformationen in sozialen Medien besser zu verstehen sowie ihre Qualität und konstruktive Nutzung zu fördern.

Die Onlinebefragung des Europäischen MSM Internet Survey (EMIS) bildet für den Beitrag von *U. Marcus *und* S. B. Schink* die Grundlage zur Beschreibung des Sexualverhaltens von Männern, die Sex mit Männern haben (MSM). Dies ist für die Planung von HIV- und STI-Präventions- und -Behandlungsprogrammen von besonderer Relevanz.

Die beiden nachfolgenden Publikationen thematisieren neben anderen Fragen auch den Einfluss der COVID-19-Pandemie.

*Brockmeyer et al. *untersuchen Daten zu Sexualverhalten und STI-Prävention bei Menschen mit diversen Lebenswelten, die im Versorgungszentrum für Sexuelle Gesundheit und Medizin (WIR) erhobenen wurden. Diese können nach Einschätzung der Autorinnen und Autoren für die Gestaltung von Präventionskonzepten von großer Bedeutung sein. Im Rahmen ihrer Analysen finden sie auch klare Hinweise, dass sich im Verlauf der COVID-19-Pandemie das Sexualverhalten in einzelnen Gruppen von Männern und Frauen verändert hat.

*D. Szücs et al.* fokussieren die gesundheitlichen Auswirkungen und Veränderungen der Gesundheitsversorgung von trans Personen während der COVID-19-Pandemie auf der Grundlage einer Onlinequerschnittstudie in deutschsprachigen Ländern. Die Ergebnisse weisen darauf hin, dass bisherige Vulnerabilitäten für gesundheitliche Probleme durch die Pandemie verstärkt wurden und der Zugang zu einer qualifiziert informierten Trans-Gesundheitsversorgung eingeschränkt wurde.

Wir hoffen, dass wir Ihnen mit dem Themenheft ein breites und interessantes Spektrum der aktuellen sexualwissenschaftlichen Forschung zusammengestellt haben. Wir wünschen Ihnen, liebe Leserinnen und liebe Leser, eine spannende Lektüre.
